# Synergistic action of cisplatin and echistatin in MDA-MB-231 breast cancer cells

**DOI:** 10.1007/s11010-016-2894-8

**Published:** 2016-12-19

**Authors:** Robert Czarnomysy, Arkadiusz Surażyński, Bożena Popławska, Edyta Rysiak, Natalia Pawłowska, Anna Czajkowska, Krzysztof Bielawski, Anna Bielawska

**Affiliations:** 1 Department of Synthesis and Technology of Drugs, Medical University of Bialystok, Kilinskiego 1, 15-089 Bialystok, Poland; 2 Department of Medicinal Chemistry, Medical University of Bialystok, Kilinskiego 1, 15-089 Bialystok, Poland; 3 Department of Biotechnology, Medical University of Bialystok, Kilinskiego 1, 15-089 Bialystok, Poland

**Keywords:** Breast cancer cells, Cisplatin, Echistatin, Apoptosis, Cell signaling

## Abstract

The aim of our study was to determine whether the use of cisplatin in the presence echistatin in MDA-MB-231 breast cancer cells leads to a reduction of toxic effects associated with the use of cisplatin. The expression of β_1_-integrin and insulin-like growth factor 1 receptor (IGF-IR), signaling pathway protein expression: protein kinase B (AKT), mitogen-activated protein kinases (ERK1/ERK2), nuclear factor kappa B (NFκB), and caspase-3 and -9 activity was measured after 24 h of incubation with tested compounds to explain detailed molecular mechanism of induction of apoptosis. The viability of MDA-MB-231 breast cancer cells was determined by 3-(4,5-dimethylthiazol-2-yl)-2,5-diphenyltetrazolium bromide assay. Annexin V-FITC/propidium iodide staining assay was performed to detect the induction of apoptosis. Inhibition DNA biosynthesis was determined by [^3^H]thymidine incorporation into DNA. The expression of of β_1_-integrin, IGF-IR, AKT, ERK1/ERK2, NFκB, caspase-3 and -9 was evaluated using Western blot. The results suggest that treatment of MDA-MB-231 breast cancer cells for 24 h cisplatin plus echistatin severely inhibits cell growth and activates apoptosis by upregulation of caspase-3 and -9 expressions. The effect was stronger than treatment cisplatin and echistatin alone. In this study, we have found that cisplatin plus echistatin treatment decreases collagen biosynthesis in MDA-MB-231 breast cancer cells stronger than the individual compounds. The inhibition was found to be dependent on the β_1_-integrin and IGF receptor activation. A significant reduction of ERK1/ERK2, AKT expression in cancer cells after cisplatin plus echistatin treatment was also found. The cancer cells treated by echistatin, cisplatin, and in particular the combination of both compounds drastically increased expression of NFκB transcription factor. Our results suggest that combined therapy cisplatin plus echistatin is a possible way to improve selectiveness of cisplatin. This mechanism probably is due to downregulation of expression of β_1_-integrin and IGF-IR receptors, and the signaling pathway proteins induced by these receptors. Our results suggest that therapy cisplatin plus echistatin is a possible way to improve selectiveness of cisplatin.

## Introduction

Breast cancer is one of the most frequently diagnosed cancers in females [1]. Most cases of this disease occur in women at the age over 60. Despite significant progress toward understanding the mechanism of the disease, effective treatment is still lacking. The cornerstone treatment for numerous malignancies, including breast cancer is cisplatin [2]. Despite its success, the clinical usefulness of cisplatin is limited by its severe side effects such as neurotoxicity, myelosuppression, nephrotoxicity, and hepatotoxicity [3–6]. The need for alternatives to cisplatin has consequently inspired further work toward the development of novel platinum-based drugs or combined therapy with other components [7–11]. Combined therapy with e.g., anthracyclines, platinum compounds, and taxanes produces higher response rates, although combination regimens have not always improved survival rate. Doxorubicin plus cisplatin has been accepted as the Gynecologic Oncology Group standard regimen based on phase III clinical data [2].

Integrins are family of adhesive receptors that are responsible for recognition and adhesion of cells to extracellular matrix proteins [12]. The interaction between integrin receptors and extracellular matrix proteins, e.g., collagen is implicated in regulation of cellular gene expression, differentiation, and cell growth [13]. Integrin receptor signaling can play an important role in tumorigenicity and invasiveness [14]. Therefore, modulation of these receptors by disintegrins or intergin activators may represent potential strategy for cancer therapy. Disintegrin can interfere in important processes involved in carcinogenesis, tumor growth, invasion, and migration [15]. Additionally, in vivo co-administration disintegrins with cancer cells markedly inhibited tumor growth and bone destruction. Moreover, it has been shown that these proteins are capable to interact with specific integrins and to inhibit their activity [16]. These observations prompted the exploration of pharmacological blockade of integrins which was eventually demonstrated to significantly reduce tumor angiogenesis in numerous cancer models including breast cancer. Among of the breast cancer cell lines, most integrin receptors have MDA-MB-231 cell line. Other breast cancer cell lines are deficient phenotype of integrin receptors, e.g., MCF-7, MDA-MB-436, and MDA-MB-468 lack α6β4 integrin receptor [17].

The disintegrins ability to inhibit cell–matrix and cell–cell interactions has been considered in many aspects. They were found to be the potent inhibitors of platelet aggregation, acting through the blockade of fibrinogen binding to platelet glycoprotein IIb/IIIa [18–21]. One of the main representatives of disintegrin is echistatin. It is known that echistatin as an inhibitor of β_1_-integrin receptor contributes to inhibition of collagen biosynthesis and decrease in the expression of FAK (focal adhesion kinase), SOS-protein (son of sevenless protein), and phosphorylated MAP-kinases (mitogen-activated protein kinases), and ERK1 (extracellular signal-regulated kinase 1) and ERK2 (extracellular signal-regulated kinase 2) [18]. Stimulated β_1_-integrin receptor induces autophosphorylation of non-receptor protein kinase FAK, which is then capable of interacting with adaptor proteins, such as Grb2 (growth factor receptor bound protein 2), through Src and Shc proteins. This interaction allows activating further cascade of signaling pathway through SOS, Ras, and Raf proteins and subsequently, two MAP-kinases: ERK1 and ERK2. The end point of this cascade is induction of transcription factor(s) that regulate(s) gene expression of integrins, proteinases, and many proteins involved in the regulation of cell growth and differentiation [22–24].

The present study was undertaken to evaluate the effect of combined treatment of cisplatin plus echistatin on expression of some signaling proteins (EKR1/ERK2, AKT), transcription factors (NFκB), receptors (β_1_-integrin, IGF-I), as well on apoptosis and cell growth in MDA-MB-231 breast cancer cells. The results from combined treatment were compared with those obtained using monotherapy.

## Materials and methods

### Chemical

Cisplatin, echistatin, DMSO, 3-(4,5-dimethylthiazole-2-yl)-2,5-diphenyltetrazolium bromide (MTT), sodium dodecyl sulfate (SDS) were provided by Sigma-Aldrich (USA). Stock culture of human MDA-MB-231 breast cancer was purchased from the American Type Culture Collection (USA). Dulbecco’s minima essential medium (DMEM) and fetal bovine serum (FBS) used in a cell culture were products of Gibco (USA). Glutamine, penicillin, and streptomycin were obtained from QualityBiological Inc. (USA). [^3^H]thymidine (6.7 Ci/mmol) were purchased from NEN (USA), and scintillation cocktail “Ultima GoldXR” from Packard (USA). FITC Annexin V Apoptosis Detection Kit was a product of BD Pharmigen (USA).

### Cell culture

Human breast cancer MDA-MB-231 maintained in DMEM supplemented with 10% fetal bovine serum (FBS), 50 U/mL penicillin, 50 µg/mL streptomycin at 37 °C in 5% CO_2_. Cells were cultured in Costar flasks, and subconfluent cells were detached with 0.05% trypsin and 0.02% EDTA in calcium-free phosphate buffer red saline, counted in hemocytometers, and plate at 5 × 10^5^ cells/well of 6-well plate (Nunc) in 2 mL of growth medium. Cells reached about 80% of confluency at day 3, and most such cells were used for the assays.

### Cell viability assay

The assay was performed according to the method of Carmichael et al. [25], using 3-(4,5-dimethylthiazole-2-yl)-2,5-diphenyltetrazolium bromide (MTT). Confluent cells, cultured for 24 h with various concentrations of studied compounds in 6-well plates were washed three times with PBS and incubated for 4 h in 1 mL of MTT solution (5 mg/mL of PBS) at 37 °C in 5% CO_2_ in an incubator. The medium was removed and 1 mL of 0.1 mol/L HCl in absolute isopropanol was added to the attached cells. Absorbance of converted dye in living cells was measured at a wavelength of 570 nm. Cell viability of breast cancer cells cultured in the presence of ligands was calculated as percent of control cells.

### Flow cytometry assessment of Annexin V binding

Apoptosis was determined using the assessment of phosphatidylserine exposure by Annexin V-FITC binding by means of the Annexin V-FITC staining kit (FITC Annexin V Apoptosis Detection Kit, BD Pharmigen, USA) according to the manufacturer’s instruction. Ungated cells (10,000 cells measured) were analyzed in a flow cytometer (FACSCanto II, BD, USA). Annexin V bound with affinity to phosphatidylserine and thus could be used to identify cells in all stages of the programmed cell death [26, 27]. Propidium iodide (PI) exclusively stained cells with a disrupted cell membrane and could be used to identify late apoptotic and dead cells. Cells were incubated with various concentrations of studied compounds for 24 h. The cells were trypsinized and resuspended in DMEM. After that time, the cells were suspended in binding buffer for staining with FITC-Annexin V and PI for 15 min at room temperature in the dark, following the manufacturer’s instructions. Cells cultured in a drug-free medium were used as controls. Optimal parameter settings were found using a positive control (cells incubated with 3% formaldehyde in buffer during 30 min on ice). Data were analyzed with FACSDiva software (BD Bioscences Systems, USA).

### [^3^H]thymidine incorporation assay

Cells were seeded in 6-well plates and grown as described above. Cells culture were incubated with varying concentrations of echistatin, cisplatin, and 0.5 Ci/mL of [^3^H]thymidine for 4 h at 37 °C. After that time, the cells surface was rinsed two times with 1 mL of 0.05 M Tris–HCl (pH 7.4) containing 0.1 M NaCl and two times with 1 mL of 5% TCA. Then the cells were lysed in 1 mL of 0.1 M NaOH containing 1% SDS. The cell lysate was added to 2 mL of scintillation liquid, and radioactivity incorporation into DNA was measured in a scintillation counter.

### 5-[^3^H]proline incorporation assay

Cells were labeled for 24 h with the 5-[^3^H]proline (5 μCi/mL, 28 Ci/mmol). Its incorporation into proteins was measured as described previously [28]. Incorporation of the tracer into collagen was determined by digesting proteins with purified *Clostridium histolyticum* collagenase, according to the method of Peterkofsky et al. [29]. The results are shown as combined values for the cell plus medium fractions.

### Western blot analysis

Samples of the lysates containing 25 μg of protein were subjected to SDS-PAGE electrophoresis, as described by Laemmli [30]. Electrophoresis was run for 60 min using a 7.5% polyacrylamide gel, and constant current of 25 mA was applied. The resolved proteins were transferred to nitrocellulose membranes and pre-incubated with Tris-buffered saline (TBS) containing 0.05% Tween 20 (TBS-T) and 5% non-fat dry milk for 2 h. Membranes were soaked in a mixture of monoclonal anti-phospho-IGF-I antibody (1:1000), monoclonal antibody β_1_-integrin (1:1000), monoclonal anti-phospho-MAPK antibody (ERK1/ERK2) (1:1000), monoclonal anti-phospho-AKT antibody (1:1000), polyclonal NFκB antibody (1:1000), polyclonal caspase-9 antibody (1:1000), polyclonal caspase-3 antibody (1:1000) in 5% dried milk in Tris-buffered saline with Tween 20 (TBS-T). Next, 1 h incubation with secondary alkaline phosphatase-conjugated antibody against rabbit or mouse IgG at the 1:5000 dilution was carried out. Finally, the nitrocellulose membranes were washed five times with TBS-T and exposed to Sigma-Fast BCIP/NBT reagent

### Statistical analysis

All numerical data are presented as mean ± standard deviation (SD) from at least three independent experiments. Statistical analysis was conducted using the Origin 7.5 software (OriginLab, USA). Statistical differences in multiple groups were determined by one-way ANOVA followed by Tukey’s test. *p* < 0.05 and *p* < 0.01 were considered statistically significant.

## Results

**Fig. 1:**
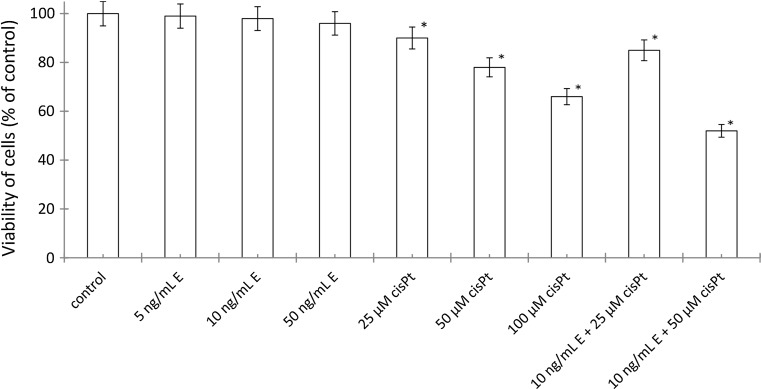
Viability assay according to the method of Carmichael et al. of MDA-MB-231 breast cancer cells treated for 24 h with various concentrations of echistatin (E), cisplatin (cisPt), or cisplatin plus echistatin. Mean values ±SD from three independent experiment (*n* = 3) done in duplicate are presented. *p < 0.05 versus control group

To evaluate cytotoxicity of echistatin, cisplatin, and cisplatin plus echistatin, the viability of breast cancer MDA-MB-231 cells was measured by the method of Carmichael et al. [25]. Incubation of the cells for 24 h with disintegrin at concentrations 5, 10, and 50 ng/mL of medium had no significant effect on the cell viability (Fig. 1). Cisplatin at concentrations 25, 50, and 100 μM induced the decrease of the cell viability to 90, 78, and 66% of control value, respectively (Fig. 1). However, incubation of MDA-MB-231 cells with 10 ng/mL of echistatin with 25 or 50 μM cisplatin decreased the cell viability to 85 and 52%. Combination of those components decreased viability more effective than the cells were treated with disintegrin or cisplatin alone in the same concentration (Fig. 1).

**Fig. 2:**
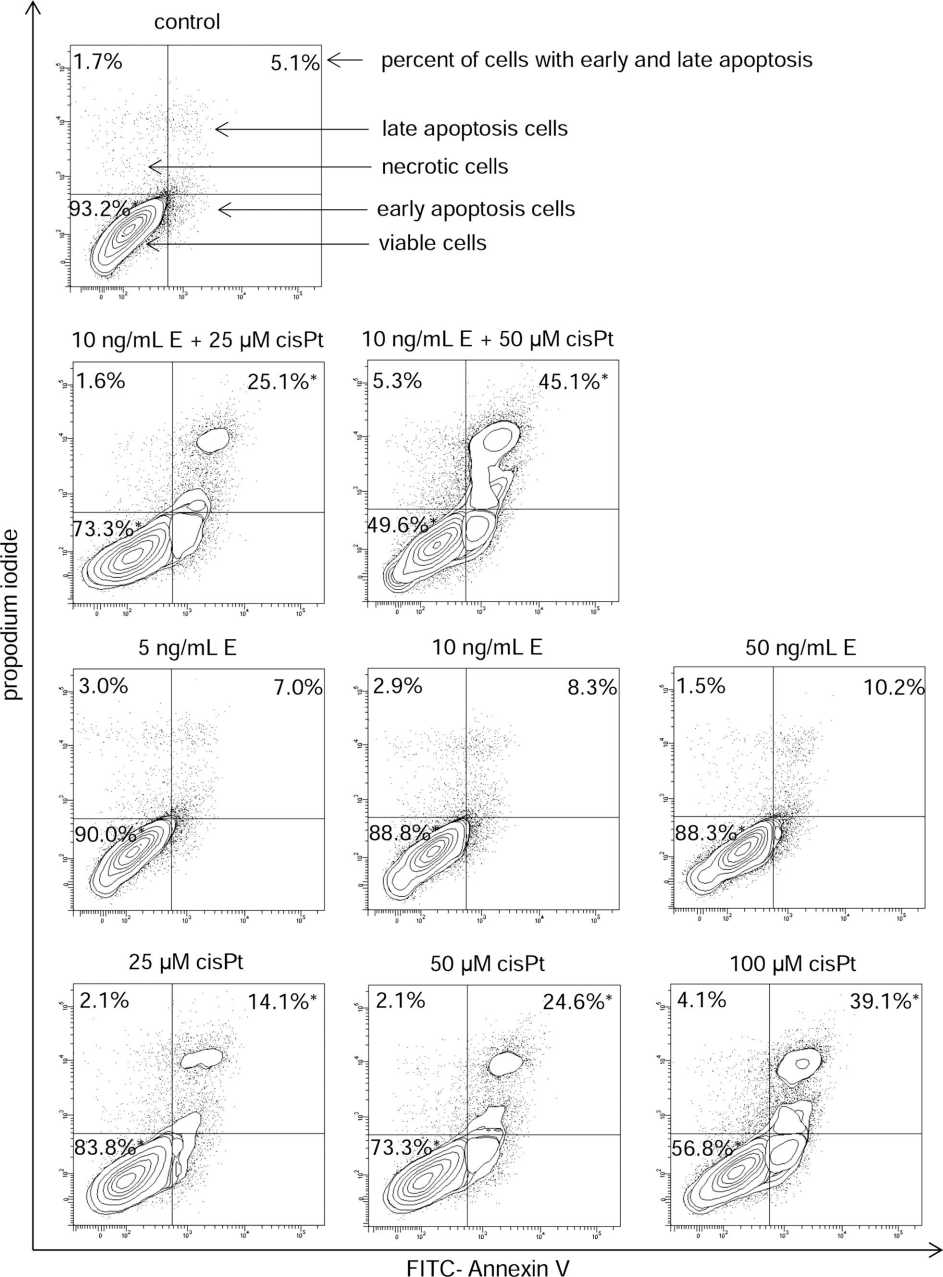
Flow cytometer analysis of MDA-MB-231 breast cancer cells after incubation with of echistatin (E) and cisplatin (cisPt), or cisplatin plus echistatin for 24 h and subsequent staining with Annexin V and propidium iodide (PI). We presented representative dot plots form one of three independent experiments. Mean percentage values from three independent experiments (*n* = 3) done in duplicate are presented. *p < 0.05 versus control group

In order to evaluate whether echistatin, cisplatin, and cisplatin plus echistatin triggered apoptosis in the breast cancer cells, the cell death was measured by flow cytometer analysis after Annexin V-FITC and propidium iodide staining. The incubation of MDA-MB-231 cells with echistatin at concentrations 5, 10 and 50 ng/mL of medium had no significant effect on the cell apoptosis (Fig. 2). Cisplatin at concentrations 25, 50, and 100 μM induced increase of the cell apoptosis about 16.1, 24.6, and 39.1% of control value, respectively. Incubation of MDA-MB-231 cells with 10 ng/mL of echistatin and 25 or 50 μM cisplatin significantly increased the cell apoptosis after 24 h of treatment about 25.1 and 45.1% of control value (Fig. 2).

**Fig. 3:**
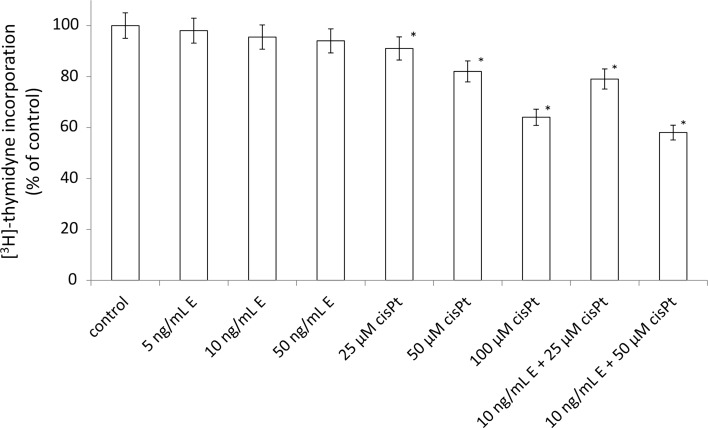
DNA biosynthesis, measured by [^3^H]-thymidyne incorporation into DNA in breast cancer cells MDA-MB-231. Cells were incubated for 24 h with various concentrations of echistatin (E), cisplatin (cisPt), or cisplatin plus echistatin. Mean values ±SD from three independent experiment (*n* = 3) done in duplicate are presented. *p < 0.05 versus control group

**Fig. 4:**
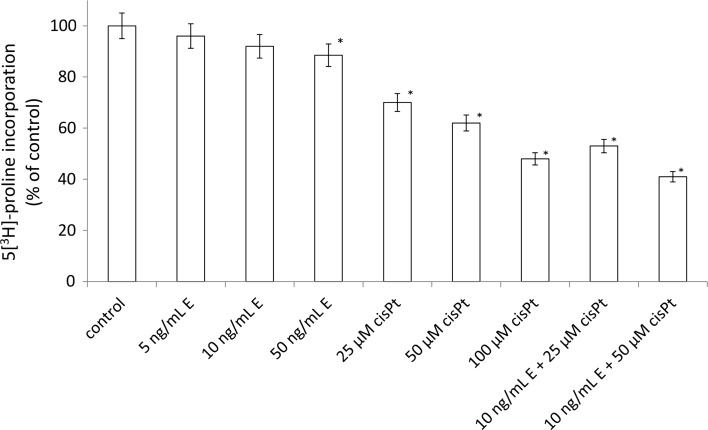
Collagen biosynthesis measured as 5[^3^H]-proline incorporation into proteins susceptible to the action of bacterial collagenase in MDA-MB-231 breast cancer cells incubated for 24 h with various concentrations of echistatin (E), cisplatin (cisPt), or cisplatin plus echistatin. Mean values ±SD from three independent experiment (*n* = 3) done in duplicate are presented. *p < 0.05 versus control group

To further investigate the possible mechanism responsible for the growth inhibitory effects, we measured DNA synthesis in the presence of the disintegrin, cisplatin, and combination of these components. Echistatin had no significant effect on the DNA biosynthesis (Fig. 3). The incubation with cisplatin at concentration 25, 50, and 100 μM decreased DNA biosynthesis about 91, 82, and 64% respectively. However, echistatin at concentration 10 ng/mL plus 50 μM cisplatin significantly decreased DNA biosynthesis about 58% of control value (Fig. 3). Similar effect was observed in respect to collagen biosynthesis. The inhibition of the protein biosynthesis was the most effective when the cells were treated with 50 μM cisplatin plus 10 ng/mL echistatin (Fig. 4).

**Fig. 5:**
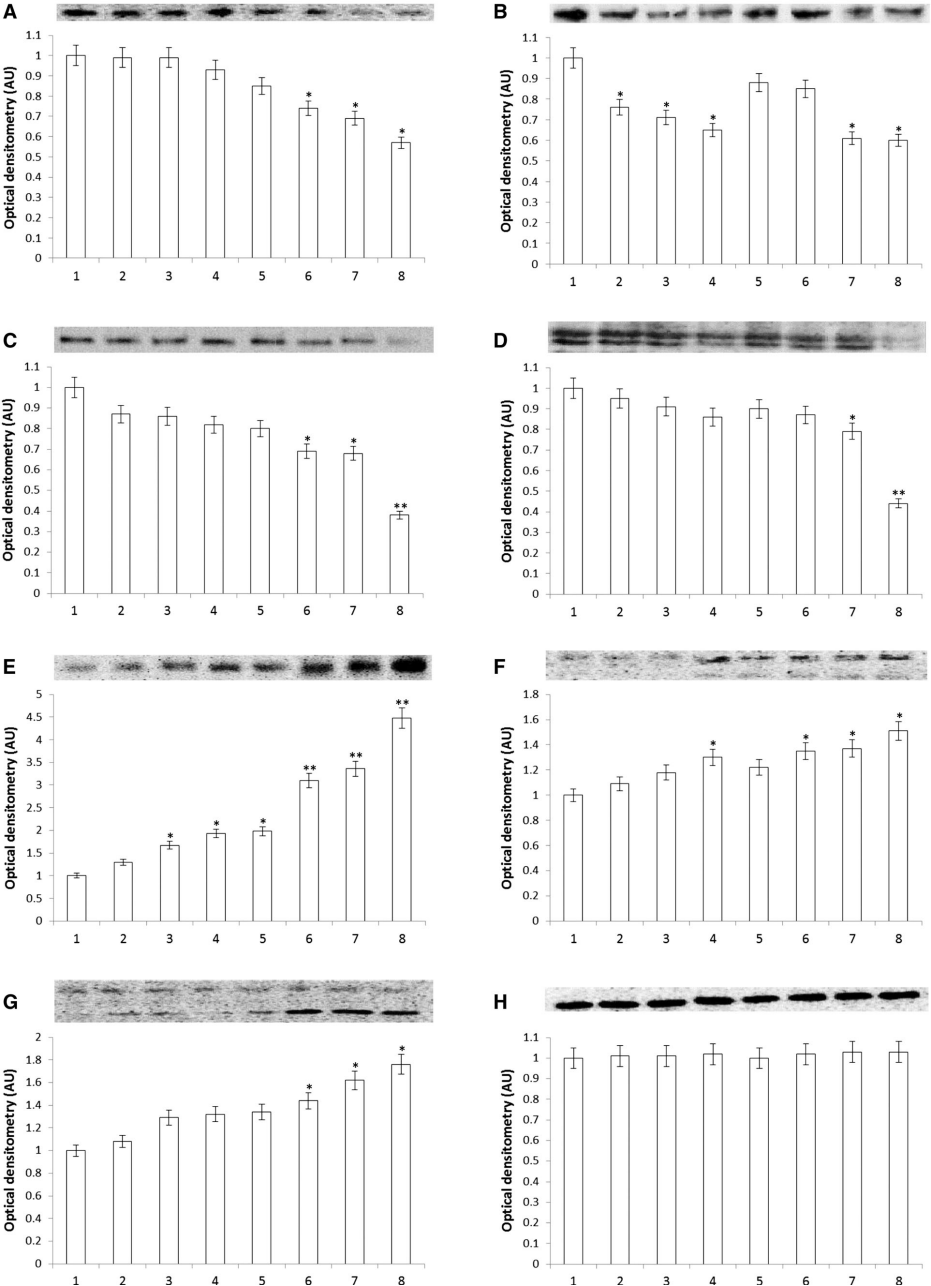
Western immunoblot analysis for IGF-IR (**A**), β_1_-integrin (**B**), AKT kinase (**C**), ERK1/2 (**D**), NFκB (**E**), caspase-9 (**F**), caspase-3 (**G**), and β-actin (**H**) in subconfluent human breast cancer cells MDA-MB-231 (control), cells treated with echistatin, cisplatin and cisplatin + echistatin for 24 h. The intensity of the bands staining was quantified by densitometry analysis. *p < 0.05 and *p < 0.01 versus control group. *1* control, *2* 5 ng/mL echistatin, *3* 10 ng/mL echistatin, *4* 50 ng/mL echistatin, *5* 25 μM cisplatin, *6* 50 μM cisplatin, *7* 10 ng/mL echistatin +25 μM cisplatin, *8* 10 ng/mL echistatin +50 μM cisplatin. Samples used for electrophoresis consisted of 25 µg protein of pooled cell extracts. *AU* arbitrary units, *IGF-IR* insulin-like growth factor 1 receptor, *AKT* protein kinase B, *ERK* extracellular signal-regulated kinase, *NFκB* nuclear factor kappa B

Cell survival, adhesive, and protein biosynthesis (e.g., collagen) are regulated by the signal mediated by activated β_1_-integrin and IGF-I receptors. The expression of the receptors was measured by Western immunoblot using specific antibodies. As shown in Fig. 5, cells with echistatin at concentrations 5, 10, and 50 ng/mL had no significant effect on the expression on IGF-IR. However, echistatin decreased β_1_-integrin receptor in dose-dependent manner. Cisplatin at concentrations 25 and 50 μM decreased expression on IGF-IR and had no effect on the expression on β_1_-integrin receptor (Fig. 5). Incubation of MDA-MB-231 cells with 10 ng/mL of echistatin and 25 or 50 μM cisplatin significantly decreases both receptors compare to control (Fig. 5).

ERK1/2 and AKT, induced by activated by β_1_-integrin and IGF-I receptor, play a central role in carcinogenesis and tumor progression. As shown in Fig. 5, expression of both kinases ERK1/2 and AKT reflects expression of β_1_-integrin and IGF-I receptor. Inhibition of those kinases may lead cells to activation of apoptosis.

We have found that cancer cells treated by echistatin, cisplatin, and in particular the combination of both compounds drastically increased expression of NFκB transcription factor, responsible among other things for collagen inhibition and apoptosis (Fig. 5).

We found also that apoptosis induced by cisplatin plus echistatin is associated with activation of caspase-9 and caspase-3, respectively. Both caspases are activated in dose-dependent manner for treatment echistatin or cisplatin. However, we observed a significant increase in expression of caspase-9 and -3 after the treatment with combination of both compounds, compared with these compounds either alone.

## Discussion

Cisplatin is one of the most widely used and effective anticancer agents. It plays a major role in the treatment of a variety of cancers, including breast, ovarian, head and neck, cervical, lung, and colorectal cancer [31, 32]. The antitumor properties of cisplatin were discovered in the 60’s by Rosenberg et al., and its clinical use was approved by the FDA in 1978. When DNA reacts with cisplatin, cross-linked adducts are produced, resulting in the distortion of its double-helix structure [33]. The efficacy of cisplatin in cancer treatment is often limited due to significant side effects and development of drug resistance. Combination of cisplatin with other drugs has been proven to be a successful therapeutic approach [3, 30]. Special emphasis has recently been given to the co-administration of cisplatin with drugs specifically targeting cancer cells [34, 35]. For example, the combination of cisplatin and trastuzumab, an antibody raised against the EGF receptor subtype HER2, has shown promising clinical activity in patients with advanced breast cancer that overexpress HER2 [34, 35]. The addition of bevacizumab, a monoclonal antibody against vascular endothelial growth factor (VEGF), to platinum-based chemotherapy resulted in an improved response and survival in patients with non-small cell lung cancer. Moreover, selenium and cisplatin interact on the same intracellular toxic cascade, and the combination of these two drugs can result in a remarkable anticancer effect through modulation of the TRPV1 [36].

Echistatin and other disintegrins have been extensively studied in the past. It is now established that disintegrins are effective agents in limiting tumor growth and spread [19, 20]. Disintegrins interacting with several integrins block various integrin-mediated processes involved in tumor growth, metastasis, and angiogenesis [21]. The integrins family is responsible for adhesion of cells to extracellular matrix components as well as to other cells [12]. The interaction affects cytoskeleton organization, lipid metabolism, kinase activation, gene expression [37], cell cycle progression [38], and metastasis [39]. Integrins β_1_ and β_3_ play also an important role in tumor invasion [40]. Cell migration, invasion, matrix degradation, proliferation, and angiogenesis are all mediated by integrins and integrin signaling effects on DNA biosynthesis [41, 42]. Moreover, the previous studies have reported that disintegrins may be reduced the protection of cancer cells from apoptosis [43]. In recent years, combined treatment of conventional anticancer agents with natural compounds (like disintegrins) has been a focus of study due to the fact that natural compounds are multi-targeted compared with designed mono-target agents and hence can overcome intrinsic cancer cell resistance to apoptosis [44]. As shown by studies of lung cancer therapy, a combined treatment with disintegrin caused a greater apoptotic effect than in its absence [45]. The same conclusions regarding combination therapy with disintegrin presented the researchers in the case hepatocellular carcinoma [46]. We found that cisplatin plus echistatin increases the sensitivity of MDA-MB-231 breast cancer cells to death through an apoptotic mechanism as compared to the use cisplatin alone. The apoptotic effect due to treatment breast cancer cells with cisplatin plus echistatin required low doses of those reagents which were compared with those obtained using monotherapy.

The higher cytotoxicity of combined treatment is also associated with a significant inhibition of DNA biosynthesis. The functional significance of the treatment of MDA-MB-231 breast cancer cells cisplatin plus echistatin was found also at the level of collagen biosynthesis. Our findings are consistent with the previous work which demonstrated that combination therapy with cytostatic agents from the group of alkylating agents and echistatin, produces a greater decrease in collagen biosynthesis than treatment with the alkylating drug alone [47]. Decreased amount of collagen in extracellular matrix may facilitate motility and invasion of neoplastic cells, but also may suppress cell growth and induce of apoptosis [48] as confirmed our results.

The previous studies have shown that IGF-I receptor signaling and β_1_-integrin receptor are involved in signaling that regulates collagen biosynthesis [49, 50]. Prokop et al. suggest that IGFBP-1 (IGF binding protein 1) may affect collagen biosynthesis through downregulation of signal generated by the integrin receptor. An addition of IGF-BP1 to the medium of cultured fibroblasts contributed to decrease in collagen biosynthesis. The same effect was achieved by echistatin—β_1_-integrin blocker [51]. The endometrial cancer cells treated for 24 h with melphalan and disintegrin showed decreased expression of IGF receptor in comparison to the cells treated with both compounds separately [47]. Our study showed that treatment cisplatin plus echistatin inhibited expression of that receptor more than cisplatin alone. IGF-IR is involved in regulation of cellular growth and transformation [52]. Therefore, inhibition of the receptor expression may represent approach to the inhibition of tumor growth. Blockade of the receptor [53] or downregulation of its expression [54] reduces cancer proliferation and induces apoptosis. Decreased expression of the receptor by cisplatin plus echistatin could explain decreased biosynthesis of collagen in the cells, since IGF-IR is a most potent inducer of collagen biosynthesis [55].

NFκB is also responsible for the inhibition of collagen gene expression [56]. It is well known that NFκB affects not only the regulation of a number of genes associated with the modulation of apoptosis, but also genes responsible for immune response, encoding proteins that regulate cell proliferation or inflammation [57, 58]. NFκB regulates the transcription genes involved in cell differentiation, proliferation, and apoptosis [56]. One of the factors strongly stimulate the expression of NFκB is cisplatin [7, 59]. In the present study, we observed an interesting effect that echistatin in combination with cisplatin to a greater extent induces expression of NFκB than cisplatin. In the case of the simultaneous presence of echistatin and cisplatin increase, NFκB correlates with an increase number of apoptotic cells. It may be one of the factors which direct tumor cells to apoptosis.

The NFκB signaling cascade interacts with several parallel pathways including the signaling cascades initiated by phosphatidylinositol 3-kinase (PI3K) and AKT [60]. The AKT and MAPK are main signaling molecules correlated with the activation of the p53 pathways in different cells and tissue types [61]. AKT is known as a signaling inductor of cell survival acting through upregulation of anti-apoptotic proteins. Simultaneously, a family of serine-threonine protein kinases, MAPKs, plays significant roles in relation to the p53 pathway in different cancer cells [62]. ERKs also play key roles in cell growth and survival and are generally overexpressed in many cancer cell types [61]. Different studies have demonstrated that the anticancer effect of anticancer drugs like trastuzumab, tamoxifen, doxorubicin, and paclitaxel involved an inhibition of the MAPK and PI3K/AKT resulting in a p53-mediated apoptosis [63, 64]. In our study, the synergistic effect on cisplatin plus echistatin a much greater blocked the extracellular signal-regulated kinase (ERK1/ERK2) and AKT in MDA-MB-231 cell than cisplatin or echistatin alone in the same concentrate. Combination of cisplatin and echistatin induces increase of the expression of NFκB and decrease of the expression of AKT leads to increase apoptosis, what we observed in our study.

## Conclusions

Our results suggest that therapy cisplatin plus echistatin is a possible way to improve selectiveness of cisplatin. These results indicate that the use cisplatin plus echistatin may constitute a new strategy in chemotherapy of breast cancer.
